# Effects of acute hypothyroidism on plasma melatonin and *Aanat* and *Asmt* expression in the pineal gland and gonads of rats

**DOI:** 10.3389/fendo.2024.1322867

**Published:** 2024-08-01

**Authors:** Rafaella Valete Nunes Paiva, Pedro Henrique de Lima Mondes, Beatriz de Jesus Brandão, Julia Nascimento Sant’Anna, Maria Eduarda Freire dos Santos, Yasmin Muniz Fighera, Luciano Cardoso Santos, Regina P. Markus, Pedro Augusto Carlos Magno Fernandes, Juneo Freitas Silva, Eduardo Koji Tamura

**Affiliations:** ^1^ Chronobiology Research Group, Department of Health Sciences, State University of Santa Cruz, Ilhéus, Brazil; ^2^ Reproduction and Endocrinology Research Center, Department of Biological Sciences, State University of Santa Cruz, Ilhéus, Brazil; ^3^ Chronopharmacology Laboratory, Department of Physiology, Institute of Biosciences, University of São Paulo, São Paulo, Brazil

**Keywords:** melatonin, extrapineal melatonin, AANAT, ASMT, ovary, testicle, reproduction, endocrinopathies

## Abstract

**Introduction:**

The reproductive system is tightly regulated by environmental and physiological signals. Melatonin, known as the hormone of darkness, plays a crucial role in regulating both the circadian and reproductive systems in mammals. Hypothyroidism is a key endocrine disorder that harms the reproductive system. Despite many studies on melatonin’s effects on the reproductive system, there is conflicting information regarding melatonin synthesis modulation in hypothyroidism. The objective of this study was to investigate the modulation of plasma melatonin levels and gene expression of *Aanat* and *Asmt* in the pineal gland and gonads of rats with hypothyroidism at different times of the day.

**Methods:**

Female and male Wistar rats were divided into control and hypothyroid groups. Hypothyroidism was induced using propylthiouracil (PTU) for 15 days, rats were euthanized six hours after lights on (ZT6), before lights off (ZT11.5), and six hours after lights off (ZT18). Free thyroxine (FT4) and melatonin were quantified in plasma, and gene expressions of melatonin synthesizing enzymes (*Aanat* and *Asmt*) were measured in pineal and sexual organs (testis and ovary). Also, morphological analysis was performed in sexual organs.

**Results:**

The results reveal some disparities between the sexes. Hypothyroidism reduced antral and primary follicles in the ovary, and reduced the weight of testis, epididymis, and prostate. In relation to gene expression, we observed a reduction in *Aanat* expression in the pineal gland during the light phase (ZT6), and in males, this reduction occurred during the dark phase (ZT18). Regarding *Asmt* expression, there was a decrease in females also during the dark phase (ZT18). In the gonads, there was an increase in expression in both sexes at ZT11.5. Additionally, it was interesting to observe the association between FT4 levels and *Asmt* expression in the gonads.

**Conclusions:**

This study showed that acute hypothyroidism can affect components of the melatonergic system in gonads, particularly gene expression of melatonin synthesis enzymes (*Aanat* and *Asmt*) contributing to changes in reproduction organs during disease progression. These findings enhance our understanding of melatonin synthesis in the reproductive system during hypothyroidism, showing distinct responses in male and female rats, and suggest that hypothyroidism affects the circadian rhythmicity of melatonin synthesis in a sex-dependent manner.

## 1 Introduction

The circadian rhythm is a biological function present in physiological systems of the organisms, including the reproductive axis (1). Melatonin has been considered one of the most important drivers behind the circadian rhythm, as it plays a crucial role in synchronizing the organism with the light-dark cycle (2). Additionally, the circadian rhythmicity of melatonin influences the regulation of the mammalian reproductive system (3).

Melatonin synthesis occurs by the pineal gland during the night, specifically in pinealocytes (4). There are also extrapineal sites of melatonin synthesis (5–7). In humans and rodents, the presence of melatonin-synthesizing enzymes, arylalkylamine N-acetyltransferase (AANAT) and acetylserotonin O-methyltransferase (ASMT), has been detected in reproductive organs such as testes, ovaries, and uterus (8–10). These enzymes exhibit a 24-hour daily rhythm and reach their peak values during the dark phase (11–13). In addition to their capacity to express enzymes related to melatonin synthesis, it has been described that these organs also contain binding sites for melatonin due to the expression of melatonin receptors, MT1 and MT2 (11–14). Therefore, the presence of components of the melatonergic system in these organs suggests local influences of melatonin on processes controlling reproduction, sexual maturation, and gonadal function (15, 16).

In fact, in the female reproductive system of humans, it has been found that melatonin plays a role in folliculogenesis, modulates ovarian steroidogenesis, protects reproductive tissues against oxidative stress, delays ovarian aging, and reduces infertility rates (17–22). During pregnancy, melatonin improves embryonic development and protects both the mother and the fetus against oxidative damage (23, 24). Furthermore, there is speculation that melatonin holds therapeutic potential for the future treatment of pregnancy complications, such as preeclampsia and preterm birth in humans (25, 26). In the male reproductive system of humans, melatonin preserves developing testicular tissue and modulates endocrine activity in Leydig cells, influencing the synthesis and release of testosterone (27, 28). Additionally, melatonin influences cell proliferation and energy metabolism in Sertoli cells (29); its supplementation improves semen quality and aspects of sperm motility (30), and provides protection against oxidative stress (31).

Therefore, we emphasize the importance of investigating melatonin synthesis in the reproductive system under pathologic conditions known to affect the reproductive system. In this context, hypothyroidism is one of the primary endocrine disorders that negatively affect the reproductive system, leading to dysfunctions in humans and other animals (32–34). However, only data regarding the role of the melatonergic system in this disease are available. In humans, a study demonstrated increased plasma melatonin levels in individuals with hypothyroidism (35), while another study observed an increase only in hypothyroid women with hyperprolactinemia (36). In rats, hypothyroidism has been shown to reduce nocturnal plasma melatonin levels (37). These results underscore the importance of further investigation for a more comprehensive understanding of the interaction between endogenous melatonin and hypothyroidism.

In light of the findings established in the preceding studies and considering the necessity to investigate whether alterations occur in this system and whether they manifest in the early stages of the hypothyroid condition or during its subsequent progression, it becomes necessary to delve deeper into melatonin synthesis in the pathological condition of hypothyroidism. Therefore, the aim of this study is to assess the plasma melatonin and the gene expression of key enzymes involved in melatonin synthesis (*Aanat* and *Asmt*) in the gonads of male and female rats under the influence of acute hypothyroidism at different times of the day.

## 2 Materials and methods

### 2.1 Animals

Male (n=36) and female (n=36) adult Wistar rats (*Rattus norvegicus*) (3 months; female, 215 ± 1.05 g; male, 295 ± 5.16 g) were used. The rats were obtained from the Laboratory of Animal Breeding, Maintenance, and Experimentation (Labio) at the State University of Santa Cruz (UESC). The animals were housed in the Labio facility and kept in metal racks within plastic boxes filled with wood shavings. The temperature was maintained at 22 ± 2°C, with a 12-hour light/dark cycle (lights on at 6:00 AM, Zeitgeber time 0 or ZT0). Airflow was controlled, and the animals had *ad libitum* access to water and a commercial diet.

All animal protocols were performed in accordance with the ethical standards of the National Council on Experimental Animal Control and were approved by Ethical Committee for Animal Experimentation (license number 26/2021) of the State University of Santa Cruz.

### 2.2 Experimental design

**Figure 1:**
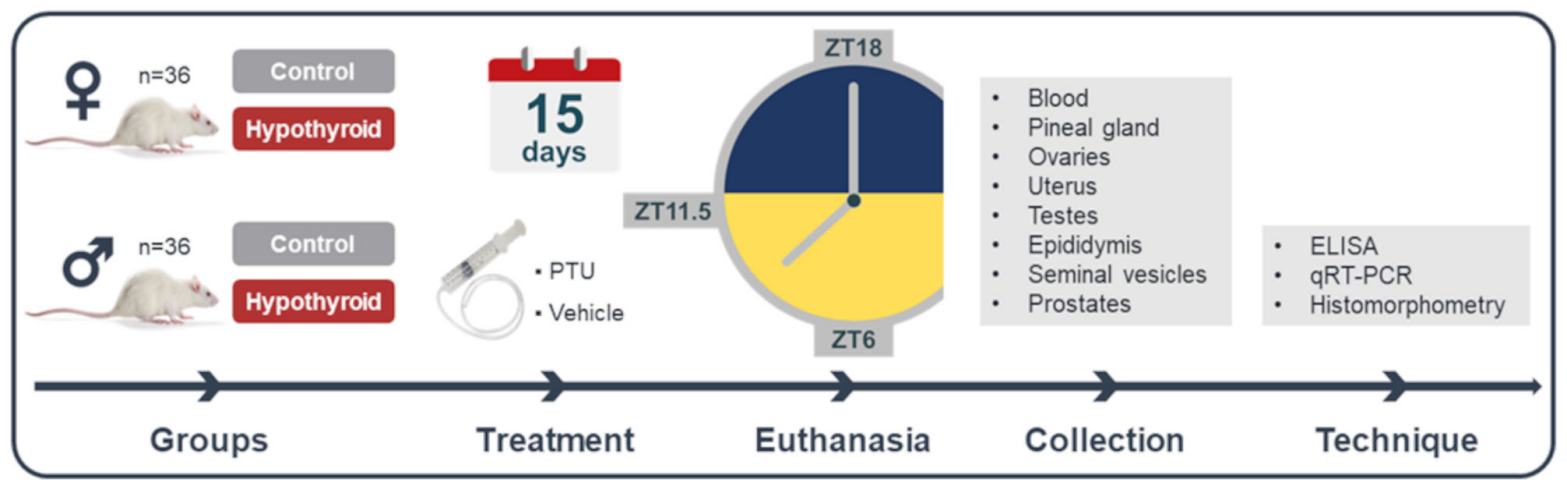
Experimental design. PTU, propylthiouracil; ZT18, 6 hours after lights off; ZT6, 6 hours after lights on; ZT11.5, before lights off.

The animals were randomly divided equally into control and hypothyroid groups, each consisting of 18 animals (**Figure 1**). Hypothyroidism was induced by daily administration of propylthiouracil (PTU, 4 mg/kg) via an orogastric tube at 8:00 AM during 15 days of treatment. The control group received water (3 mL/animal) as a vehicle (37, 38). The animals were weighed at the beginning and end of the treatment period. Euthanasia was performed by decapitation at three different time points: 6 hours after lights on (ZT6), before lights off (ZT11.5), and 6 hours after lights off under dim red light (ZT18). The females were euthanized during the proestrus phase of the estrous cycle. For this purpose, vaginal cytology was previously performed to monitor the regularity of the cycle.

After euthanasia, blood samples, pineal glands, and reproductive tract organs were collected. Plasma and pineal gland samples were collected at ZT18 under dim red light conditions. Regarding the reproductive tract organs, ovaries and uterus were collected in female rats, while testes, epididymides, seminal vesicles, and prostates were collected in male rats. After collection, all organs were weighed. The blood was used for the measurement of plasmatic free thyroxine (FT4) and melatonin levels by enzyme-linked immunosorbent assay (ELISA) method. The pineal gland, the left testis, and the left ovary were placed in microtubes containing Trizol, then frozen in liquid nitrogen and stored at -80°C for gene expression evaluation of *Aanat* and *Asmt* using reverse transcription quantitative polymerase chain reaction (RT-qPCR). The right testis, the right ovary, and uterus were used for histomorphometric evaluation.

### 2.3 Hormonal assay

The blood samples were collected from the neck using heparin tubes for the measurement of FT4 and melatonin levels. The samples were centrifuged at 3000 rpm for 20 minutes to obtain plasma, which was then stored at -20°C. The measurement of FT4 hormone was performed using an enzyme-linked immunosorbent assay (ELISA) method, using commercial kits according to the manufacturer’s instructions (IMMULITE, Siemens Medical Solutions Diagnostics, Malvern, PA, USA). The assay had a sensitivity of 0.4 ng/dL (38). The coefficients of intra-assay and inter-assay variation were determined to be 7%. Plasma melatonin was measured using an ELISA kit with a detection limit of 3.0 pg/mL (IBL, Hamburg, Germany). The plate readings were performed using a microplate spectrophotometer (SpectraMAX 250; Molecular Devices, Sunnyvale, CA, USA) at a wavelength of 405 nm (39).

### 2.4 Histomorphometry

Testes, ovaries, and uteri samples were fixed in 4% paraformaldehyde for 24 hours, followed by preservation in 70% alcohol ethanol. Transverse sections were prepared, starting with 70% alcohol and progressing to absolute alcohol. After dehydration in xylene, the samples were embedded in paraffin. 4 µm thick histological sections were obtained using a microtome (LEICA RM 2145, Germany). The slides were stained with hematoxylin and eosin (H&E). Images of the histological slides were captured using a light microscope with a camera (Leica DM 2500, Leica Microsystems, Germany), and measurements were performed using Image-Pro Plus 4.5 software (Media Cybernetics, USA). In the testis, the height and diameter of the seminiferous tubules of six animals were analyzed. Thirty transverse sections were measured for each animal, and the mean values were determined (40). In the ovaries of the six animals collected for analysis, we counted the number of primordial, secondary, antral follicles, and corpus luteum. We used a stratified sampling approach, where every fifth section of the ovaries was randomly selected to estimate the total counts of primordial, primary, preantral, antral follicles, and corpora lutea per ovary (41). For the uterus evaluation, was based on averaging the measurements of the thickness of both the endometrium and myometrium from four projections. This assessment relied on the maximum and minimum thickness observed in the sagittal section, measured in micrometers (μm) (42).

### 2.5 Real-time RT-PCR

Total RNA was extracted from the pineal glands, testes, and ovaries using the TRizol kit (Life Technologies, USA) following the manufacturer’s instructions. cDNA synthesis was performed with 1 µg of RNA using the GoScript™ Reverse Transcriptase kit (PROMEGA, USA). Gene transcript quantification was carried out using the GoTaq^®^ qPCR Master Mix on the Applied Biosystems 7500 Fast Real-Time PCR System (Applied Biosystems, Life Technologies). RT-qPCR reactions were prepared with 1.5 µL of cDNA, 1.0 µL of each primer, and 10 µL of the Master Mix reagent, in a final volume of 20 μL, following the instructions of the GoTaq^®^ qPCR and RT-qPCR Systems kit (PROMEGA, USA). Negative controls included replacing the cDNA sample with water. The amplifications were performed with the following conditions: enzymatic activation at 95°C for 2 minutes, followed by 40 cycles of denaturation at 95°C for 15 seconds and annealing/extension at 60°C for 60 seconds. Primers were designed based on the messenger RNA sequence of *Rattus norvegicus* (**Table 1**). Gene expression was calculated using the 2-ΔΔCT method, comparing the results obtained for each group after normalization with glyceraldehyde-3-phosphate dehydrogenase (*Gapdh*) expression for pineal glands and testes, and ribosomal protein L19 (*L19*) expression for ovaries (43).

**Table 1:** List of genes with primer sequences.

Gene	Primer sequences	Accession number
*Aanat*	Forward, 5′-AGCGCGAAGCCTTTATCTCA-3’ Reverse, 5′-AAGTGCCGGATCTCATCCAA-3’	NM_012818.2
			*Asmt*	Forward, 5′-AGCGCCTGCTGTTCATGAG-3’ Reverse, 5′-GGAAGCGTGAGAGGTCAAAGG-3’	NM_144759.2
*Gapdh*	Forward, 5′-TTCTTGTGCAGTGCCAGCC-3’ Reverse, 5′-GTAACCAGGCGTCCGATACG-3’	NM_017008.4			
			*L19*	Forward, 5′-CGC CAA TGC CAA CTC TCG TCA-3’ Reverse, 5′-TTC CGT CGG GCC AAA GGT GTT C-3’	NM_031103.1

### 2.6 Statistical analysis

The data are presented as mean ± standard error of the mean (SEM). The FT4 levels, body weight and sexual organ, and morphological sexual parameters data were shown as a percentage of the mean of the total control group (dependently of euthanized ZT point). All data were tested for normality deviation and sample homogeneity using the Shapiro-Wilk test. The comparison between the means of two groups was performed using the Student’s t-test for independent or paired samples. Comparisons between means of more than two groups and with two factors, treatment (Saline versus PTU) and time (ZT6 versus ZT11.5 versus ZT18) were conducted using two-way analysis of variance (two-way ANOVA), followed by the Bonferroni post-test. The correlations between different variables were performed using the Pearson correlation coefficient. Significance was always considered at p <0.05. All statistical analyses were performed using GraphPad Prism software version 8.0 (GraphPad Software, USA).

## 3 Results

### 3.1 Confirmation of hypothyroidism and evaluation of reproductive morphology

The reduction of FT4 levels by a daily (15 days) treatment with PTU confirmed the induction of hypothyroidism. Body weight, sexual organs and morphological measurements disclosed specific changes induced by hypothyroidism in the reproductive organs of female (**Supplementary Table S1**) and male rats (**Supplementary Table S2**).

**Figure 2:**
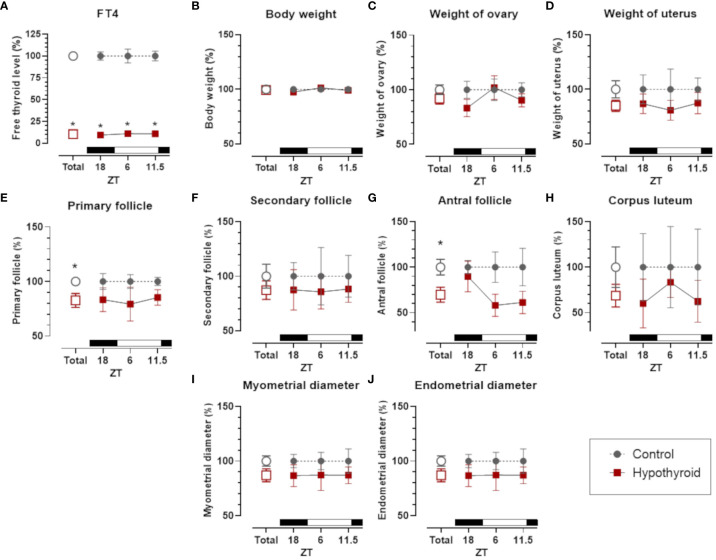
Comparison of thyroid function, body weight and sexual organs, and morphological parameters between control and hypothyroid groups of female rats. Free T4 (FT4) plasma levels **(A)**; body weight **(B)**; weight of ovary **(C)** and uterus **(D)**; number of primary follicles **(E)**, secondary follicles **(F)** antral follicles **(G)** and corpus luteum **(H)** in the ovary; diameter of the myometrium **(I)** and endometrium **(J)**. mean ± SEM; n=6 animals per group; *p<0.05 compared to the respective control group. The black bar represents the dark phase of the light-dark cycle. ZT, zeitgeber time; ZT18, 6 hours after lights off; ZT6, 6 hours after lights on; ZT11.5, before lights off.

In females, independently of the hour of the day, PTU treatment significantly reduced FT4, which did not impose a variation either in control or hypothyroid animals (**Figure 2A**). FT4 levels showed a significant interaction with the treatment factor [F (1. 5) = 531.0, p <0.0001]. Regarding body weight (**Figure 2B**) and reproductive organs, including ovaries (**Figure 2C**) and uterus (**Figure 2D**), no significant differences were observed between the control and hypothyroid groups.

The number of ovary primary follicles (**Figure 2E**) and antral follicles (**Figure 2G**) was reduced by hypothyroid group. However, no significant differences were observed in secondary follicles (**Figure 2F**) and corpus luteum (**Figure 2H**). In the histomorphometric evaluation of the uterus, no statistically significant differences were identified in the diameter of the myometrium (**Figure 2I**) and the endometrium (**Figure 2J**) between the control and hypothyroid groups.

**Figure 3:**
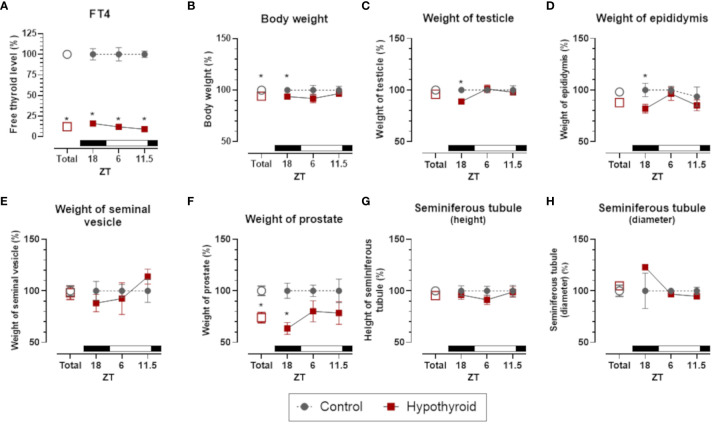
Comparison of thyroid function, body weight and sexual organs, and morphological parameters between control and hypothyroid groups of male rats. mean ± SEM; n=6 animals per group; Free T4 (FT4) plasma levels **(A)**; body weight **(B)**; weight of testicle **(C)**, epididymis **(D)**, seminal vesicle **(E)** and prostate **(F)**; seminiferous tubule height **(G)** and diameter **(H)**; *p<0.05 compared to the respective control group. The black bar represents the dark phase of the light-dark cycle. ZT, zeitgeber time; ZT18, 6 hours after lights off; ZT6, 6 hours after lights on; ZT11.5, before lights off.

In male rats, FT4 levels exhibited a significant interaction with the treatment factor [F (1. 5) = 522.6, p <0.0001], with reduced levels in the hypothyroid group compared to the control group in the overall group means. A significant reduction in FT4 levels was observed at all the hours of the day tested (ZT18, ZT6, and ZT11.5, **Figure 3A**).

It is noteworthy that in male rats the body weight and the weight of the reproductive organs (testis, epididymis, and prostate), but not the seminal vesicle, present a significant reduction only at ZT18. Thus, despite the reduction in circulating FT4, the male genitalia and the body weight maintained the same level of control at ZT6 and ZT11.5 (**Figure 3**).

Regarding histomorphometric parameters of the testes no significant difference between seminiferous tubule height (**Figure 3G**) and diameter (**Figure 3H**) were detected. Thus, despite the reduction in testes weight at ZT18, the seminiferous tubule was not affected in hypothyroid group.

### 3.2 Acute hypothyroidism modulates the melatonergic system

**Figure 4:**
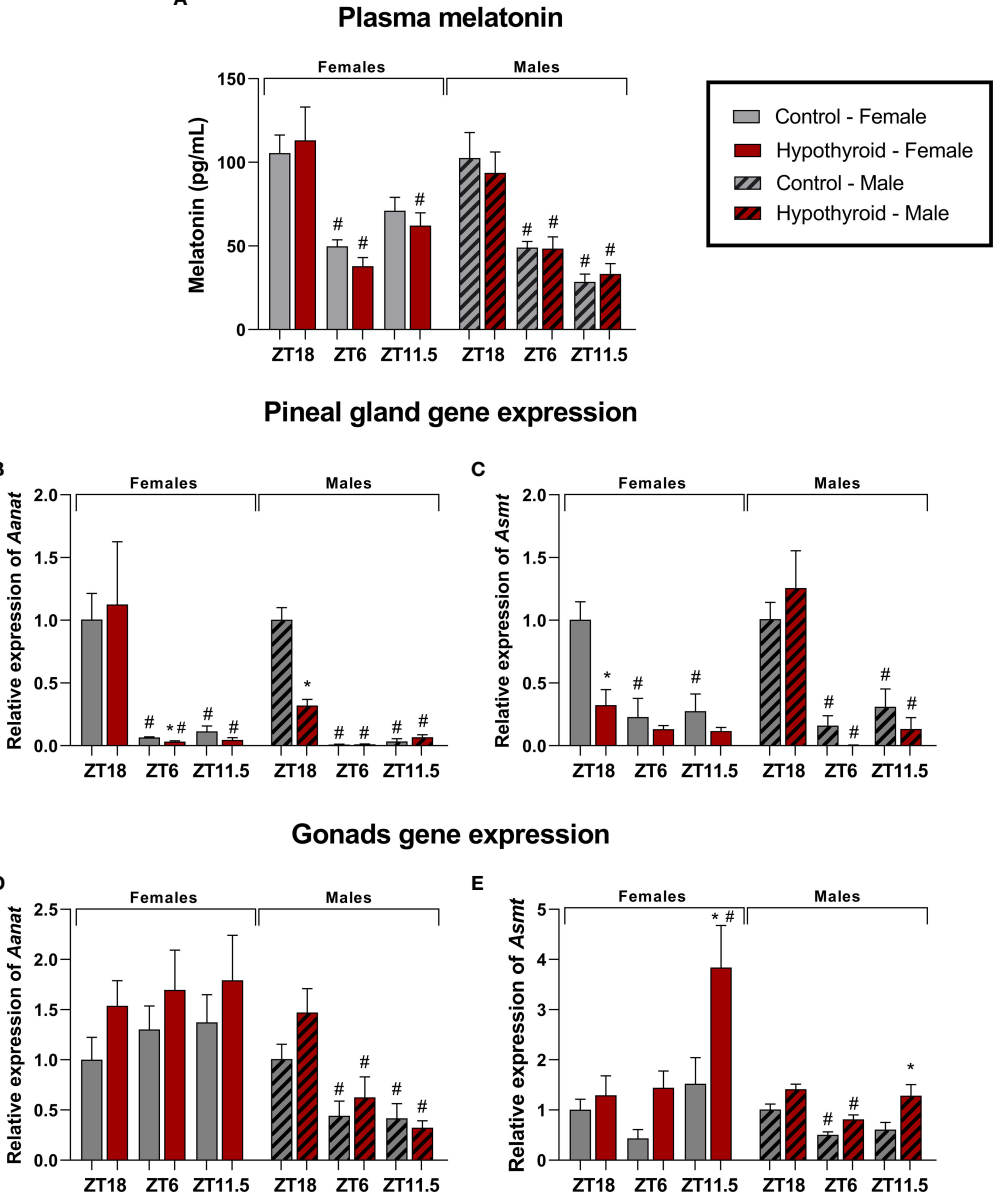
Effects of hypothyroidism on melatonin synthesis in female and male rats. Plasma melatonin levels **(A)**; Pineal gland relative gene expression of *Aanat* **(B)** and *Asmt* **(C)**; Relative gene expression of *Aanat* **(D)** and *Asmt* **(E)** in the ovaries; Relative gene expression of *Aanat* **(D)** and *Asmt* **(E)** in the testes. n = 6 animals per group. *p<0.05 compared to the respective control group; #p<0.05 compared to the ZT18 in each respective treatment; ZT18, 6 hours after lights off; ZT6, 6 hours after lights on; ZT11.5, before lights off.

To investigate the effect of acute hypothyroidism on melatonin synthesis, we assessed plasma melatonin levels and analyzed the gene expression of melatonin-synthesizing enzymes, *Aanat* and *Asmt*, in the pineal gland and gonads of both female and male rats (**Figure 4**).

In females, a significant interaction was observed for plasma melatonin levels in the time factor [F (2. 19) = 23.25, p<0.0001]. When analyzing different ZTs, the control group at ZT18 showed higher melatonin levels than ZT6 (p=0.0021). In the hypothyroid group, ZT18 animals showed higher levels compared to ZT6 (p<0.0001) or to ZT11.5 (p=0.0045), as shown in **Figure 4A**.

Significant alterations were observed in the gene expression of melatonin-synthesizing enzymes in the pineal gland. For the *Aanat* expression, significant interaction was found between the time factor [F (2. 20) = 13.32, p=0.0002]. In the control group, higher *Aanat* expression was observed at ZT18 compared to ZT6 (p=0.0230) or ZT11.5 (p=0.0198). The increase in *Aanat* expression was also observed in the hypothyroid group when compared ZT18 with ZT6 (p=0.0076) or ZT11.5 (p=0.0137). When comparing the control group and the hypothyroid group, there was decrease in *Aanat* expression, as shown in **Figure 4B**. The expression of *Asmt* in the pineal gland showed a significant interaction in the time [F (2. 10) = 12.67, p = 0.0018] and treatment [F (1. 5) = 28.90, p = 0.0030] factors. Regarding different ZTs, the control group exhibited higher *Asmt* gene expression in ZT18 than ZT6 (p=0.0004) or ZT11.5 (p=0.0008) (**Figure 4C**). Significant reduction in *Asmt* expression was observed in the hypothyroid group compared to the control group only at ZT18 (p=0.0006), as shown in **Figure 4C**.

In the ovaries, no differences were observed in *Aanat* expression (**Figure 4D**). Significant difference in *Asmt* expression was observed (**Figure 4E**), and there was an interaction between the time [F (2.10) = 5.161, p=0.0289] and treatment [F (1. 5) = 16,18, p=0.0101] factors. When comparing different ZTs, *Asmt* expression was lower at ZT 18 than ZT11.5 (p=00.54), and considering the different treatments, the hypothyroid group at ZT11.5 (p=0.0044) showed higher *Asmt* expression compared to the control group.

In male rats, plasma melatonin showed a significant interaction only in the time factor [F (2.19) = 34.48, p<0.0001]. When comparing different ZTs, the control group at ZT18 exhibited a higher melatonin level compared to ZT6 (p=0.0007) and ZT11.5 (p<0.0001). This increase was also observed in the hypothyroid group compared to ZT6 (p=0.0058) and ZT11.5 (p=0.0002), as shown in **Figure 4A**.

In the pineal gland, the expression of *Aanat* showed a significant interaction between the factors time [F(1.112, 16.67) = 129.1, p<0.0001], treatment [F (1. 30) = 34.02, p<0.0001], and time x treatment [F (2. 30) = 39.55, p<0.0001]. In the analysis of different ZTs, the control group showed higher expression of *Aanat* at ZT18, both compared to ZT6 (p=0.0005) and ZT11.5 (p=0.0008). In the hypothyroid group, there was also an increased expression at ZT18 compared to ZT6 (p=0.0042) and ZT 11.5 (p=0.0148). When comparing the treatments, the hypothyroid group exhibited a reduction in *Aanat* expression at ZT 18 (p<0.0001) compared to the control group, as illustrated in **Figure 4B**. Regarding the expression of *Asmt* in pineal gland, a significant interaction was observed between the factors time [F (2. 10) = 23.39, p=0.0002]. In the analysis of different ZTs, the control group showed higher expression of *Asmt* at Z18, compared to ZT6 (p=0.0084) and ZT11.5 (p=0.0266). It was also observed in the hypothyroid group at ZT6 (p=0.0009) and ZT11.5 (p=0.0012), as illustrated in **Figure 4C**. No differences were found between hypothyroid and control groups (**Figure 4C**).

In the testis, the expression of *Aanat* showed a significant interaction in the time factor [F (2. 10, 9.223), p=0.0054]. At different ZTs, the hypothyroid group exhibited higher expression at ZT18 compared to ZT6 (p=0.0437) or ZT11.5 (p=0.0348), as shown in **Figure 4D**. In the expression of *Asmt* in the testicles, a significant interaction was observed to the factors time [F (2. 10) = 13.13, p=0.0016] and treatment [F (1. 5) = 19.21, p=0.0071]. In the analysis of different ZTs, the control group showed higher expression of *Asmt* at ZT18 compared to ZT6 (p=0.0359). Additionally, in the hypothyroid group, an increase in expression at ZT18 compared to ZT 6 (p=0.0035) was also observed. When comparing the treatments, an increase in *Asmt* expression was observed in the hypothyroid group at ZT11.5 (p=0.0106) compared to the control group, as shown in **Figure 4E**.

### 3.3 Acute hypothyroidism and melatonergic system parameters correlations

**Figure 5:**
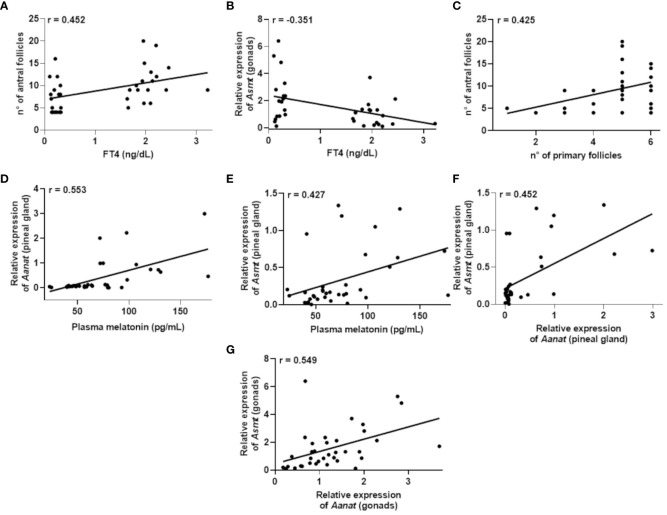
Correlations that exhibited significance (P<0.05) in the Pearson’s coefficient (r) analyses of the data obtained between two variables in female rats (n=36 animals). Correlation between: plasma Free T4 (FT4) levels and the number of antral follicles **(A)**; plasma FT4 levels and *Asmt* expression in the gonads **(B)**; number of primary follicles and antral follicles **(C)**; plasma melatonin levels and *Aanat* expression in the pineal gland **(D)**; plasma melatonin levels and *Asmt* expression in the pineal gland **(E)**; expression of *Aanat* and *Asmt* in the pineal gland **(F)**; expression of *Aanat* and *Asmt* in the gonads **(G)**.

**Figure 6:**
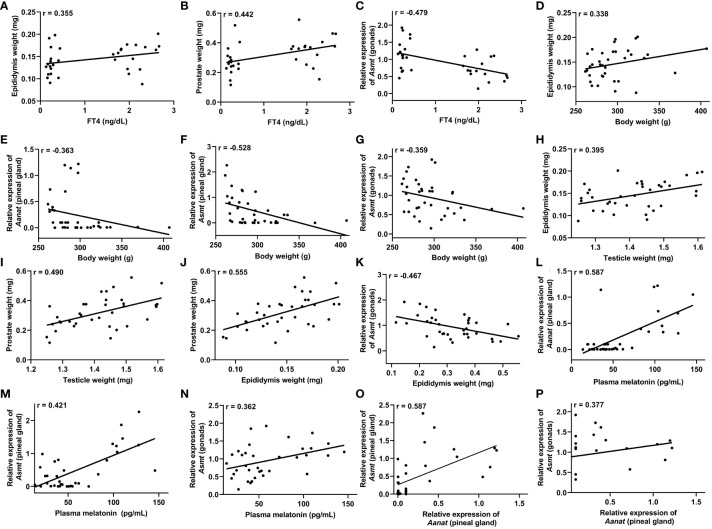
Correlations that exhibited significance (P<0.05) in the Pearson’s coefficient (r) analyses of the data obtained between two variables in male rats (n=36 animals). Correlation between: plasma free T4 (FT4) levels and the epididymis weight **(A)**, prostate weight **(B)** and *Asmt* expression in the testicles (gonads) **(C)**; body weight and epididymis weight **(D)**, *Aanat* expression in the pineal gland **(E)**, *Asmt* expression in the pineal gland **(F)** and *Asmt* expression in the gonads **(G)**; testicular weight and epididymis weight **(H)** and prostate weight **(I)**; epididymal weight and prostate weight **(J)** and *Asmt* expression in the gonads **(K)**; plasma melatonin levels and Aanat expression in the pineal gland **(L)**, *Asmt* expression in the pineal gland **(M)** and *Asmt* expression in the gonads **(N)**; *Aanat* expression in the pineal gland and *Asmt* expression in the pineal gland **(O)** and *Asmt* expression in the gonads **(P)**.

To explore potential correlations among the data obtained from the control and hypothyroid groups, we conducted a Pearson correlation analysis encompassing all animals under investigation. In this context, Pearson correlation analysis was applied to assess the linear associations between different pairs of variables. Additionally, we employed linear regression as an additional tool to comprehend underlying trends and make predictions based on the observed relationships. The outcomes of this analysis are presented in correlation tables (**Tables 2**, **3**) and regression plots (**Figures 5**, **6**).

**Table 2:** Pearson correlation among the data obtained in female rats.

	FT4	Primary Fol.	Antral Fol.	Plasma MLT	*Aanat* (pineal)	*Asmt* (pineal)	*Aanat* (ovary)	*Asmt* (ovary)
**FT4** **Primary Fol.** **Antral Fol.** **Plasma MLT** ***Aanat* (pineal)** ***Asmt* (pineal)** ***Aanat* (ovary) *Asmt* (ovary)**	1	0.227 1	0.452* 0.425* 1	-0.021 -0.017 0.130 1	-0.019 0.024 0.001 0.553* 1	0.062 0.037 0.234 0.427* 0.452* 1	-0.300 0.145 0.025 0.184 0.025 0.085 1	-0.351* 0.113 -0.115 -0.054 -0.009 0.042 0.549* 1

Values expressed in Pearson’s coefficient (r). n=36. Bold values and * denote statistical significance at the p<0.05 level. FT4, Free thyroxine; Fol., follicle; MLT, Melatonin.

**Table 3:** Pearson correlation among the data obtained in male rats.

	FT4	Body Wt.	Testicle Wt.	Epididymis Wt.	Prostate Wt.	Plasma MLT	*Aanat* (pineal)	*Asmt* (pineal)	*Aanat* (testes)	*Asmt* (testes)
**FT4** **Body Wt.** **Testicle Wt.** **Epididymis Wt.** **Prostate Wt.** **Plasma MLT** ***Aanat* (pineal)** ***Asmt* (pineal)** ***Aanat* (testes)** ***Asmt* (testes)**	1	0.322 1	0.248 0.090 1	0.355* 0.338* 0.395* 1	0.442* 0.221 0.490* 0.555* 1	0.023 -0.108 0.167 0.114 0.301 1	-0.030 -0.363* 0.008 -0.182 0.135 0.587* 1	0.185 -0.528* -0.077 -0.222 0.059 0.421* 0.587* 1	0.131 0.059 0.172 0.153 0.172 0.159 0.125 -0.149 1	**-0.479** -0.359* -0.178 -0.322 -0.467* 0.362* 0.377* 0.207 -0.005 1

Values expressed in Pearson’s coefficient (r). n=36. Bold values and * denote statistical significance at the p<0.05 level. FT4, Free thyroxine; Wt, weight; MLT, Melatonin.

Regarding females (**Table 2**), the results revealed significant correlations. There was a positive correlation between FT4 levels and the number of antral follicles, r = 0.452 (**Figure 5A**), while a negative correlation was identified between FT4 levels and *Asmt* gene expression in the gonads, r = -0.351 (**Figure 5B**). Furthermore, a positive correlation was observed between the number of primary follicles and the number of antral follicles, r = 0.425 (**Figure 5C**).

In the context of plasma melatonin levels, positive correlations were found between the expression of *Aanat* gene, r = 0.553 (**Figure 5D**) or *Asmt* gene, r = 0.427 (**Figure 5E**) in the pineal gland. Additionally, positive correlations were identified between the expression of *Aanat* and *Asmt* genes in the pineal gland, r = 0.452 (**Figure 5F**), as well as a positive correlation between the expression of *Aanat* and *Asmt* genes in the ovaries, r = 0.549 (**Figure 5G**).

In male rats, a positive correlation was observed between FT4 levels and the weight of the epididymis, r = 0.355 (**Figure 6A**), as well as with the weight of the prostate, r = 0.442 (**Figure 6B**). Additionally, a negative correlation was identified between FT4 levels and *Asmt* gene expression in the testicles, r = -0.479 (**Figure 6C**). Notably, body weight exhibited a positive correlation with epididymal weight, r = 0.338 (**Figure 6D**), but displayed negative correlations with *Aanat* gene expression, r = -0.363 (**Figure 6E**) and *Asmt* gene expression, r = -0.528 (**Figure 6F**) in the pineal gland, as well as with *Asmt* expression in the testicles, r = -0.359 (**Figure 6G**).

A positive correlation was observed between testicular weight and epididymal weight, r = 0.395 (**Figure 6H**), as well as with prostate weight, r = 0.490, (**Figure 6I**). Concerning epididymal weight, a positive correlation was found with prostate weight, r = 0.555 (**Figure 6J**). Additionally, prostate weight exhibited a negative correlation with *Asmt* gene expression in the testicles, r = -0.467 (**Figure 6K**).

Plasma melatonin levels exhibited a positive correlation with *Aanat* gene expression, r = 0.587 (**Figure 6L**) and *Asmt* gene expression, r = 0.421 (**Figure 6M**) in the pineal gland, as well as with *Asmt* expression in the testicles, r = 0.362 (**Figure 6N**). Correlations were also observed between *Aanat* gene expression in the pineal gland and *Asmt* gene expression in both the pineal gland, r = 0.587 (**Figure 6O**) and the testicles, r = 0.377 (**Figure 6P** and **Table 3**).

## 4 Discussion

This study investigated the effects of acute hypothyroidism on plasma melatonin and gene expression of biosynthetic enzymes in the melatonergic system in the pineal gland and gonads of female and male rats. The results reveal that 15 days of PTU-induced hypothyroidism promotes alteration in the gene expression of the key enzymes in the melatonergic system (**Table 4**).

**Table 4:** Effects of PTU treatment (15 days) in female and male rats.

		Females	Males
**Free thyroxine**	**ZT 18** **ZT 6** **ZT 11.5**	↓ ↓ ↓	↓ ↓ ↓
**Body weight**	**ZT 18** **ZT 6** **ZT 11.5**	= = =	↓ ↓ =
**Gonads’weight**	**Total** **ZT 18** **ZT 6** **ZT 11.5**	= = = =	↓^a^ ↓^b^ = =
**Histomorphometry**	**Total** **ZT 18** **ZT 6** **ZT 11.5**	↓^c,d^ = = =	= = = =
**Plasma melatonin**	**ZT 18** **ZT 6** **ZT 11.5**	= = =	= = =
***Aanat* (pineal gland)**	**ZT 18** **ZT 6** **ZT 11.5**	= ↓ =	↓ = =
***Asmt* (pineal gland)**	**ZT 18** **ZT 6** **ZT 11.5**	↓ = =	= = =
***Aanat* (gonads)**	**ZT 18** **ZT 6** **ZT 11.5**	= = =	= = =
***Asmt* (gonads)**	**ZT 18** **ZT 6** **ZT 11.5**	= = ↑	= = ↑

^a^effect observed only on the prostate; ^b^effect observed in the testis, epididymis, and prostate; ^c^effect observed only on the primary follicle; ^d^effect observed only on the antral follicle. ↓ decreases; ↑ increases; = no significant effect.

The FT4 hormone levels were measured to confirm the hypothyroid state, and a reduction was observed in both female and male rats in the hypothyroid group. It is important to note that PTU induction is a well-established model in animal experiments, also decreases triiodothyronine (T3) and increases thyroid-stimulant hormone (44, 45). Additionally, this drug acts by inhibiting peroxidase activity in the thyroid and peripheral deiodination of iodotyrosine, reducing the conversion of T4 to T3 (46). Although this mechanism impacts the duration and effectiveness of the treatment (15 days), as previously described in male rats, providing the experimental basis for the present study (47, 48).

Body weight reduction occurred only in male rats with hypothyroidism in the overall analysis and at specific time points of the circadian cycle (ZT18). Considering that males and females equally presented a reduction in thyroid hormone levels, we hypothesize that the PTU treatment might affect males appetite to a greater extent, especially when rodents are more active (night phase), and food consumption is higher (49, 50). In future studies, it will be interesting to measure the differences between sexes in food consumption and thyroid/melatonin responses during the induction of hypothyroidism to clarify this inquiry.

In relation to the weight of sexual organs, male rats subjected to PTU treatment showed only at ZT18 a significant reduction in the weight of the testes, epididymis, and prostate. Additionally, the prostate also exhibited a reduction in the overall analysis, suggesting that PTU affects the weight of male sexual organs in a circadian rhythmic manner. At the same time, there is no corresponding effect on female sexual organs, as sex modulates the action of thyroid hormones in an organ-specific manner (51). Therefore, the reduction observed in weight of male sexual organs in male rats subjected to PTU treatment may result from decreased thyroid hormone levels (52–57). The weight reduction only in the male sexual organs in ZT18 needs more research to elucidate the mechanism involved in this time dependent effect.

Histomorphometric analysis revealed significant changes in the ovarian follicles of hypothyroid female rats, showing decreased numbers of primary follicles and antral follicles. Hypothyroidism can affect ovarian morphology, with a reduction in the number primary follicles and pre-antral follicle, including alterations in offspring from hypothyroid pregnancies (41, 58–60). It is important to note that the females evaluated in this study were in the proestrus phase of the estrous cycle when antral follicle development and preparation for ovulation occurs (61, 62). Therefore, the findings indicate a direct impact of PTU on the morphology of ovarian follicles, negatively affecting their development and maturation and potentially compromising the ovulation and the reproductive capacity of female rats. The short duration of PTU treatment (15 days) might have limited the extent of the observed alterations. Nevertheless, the present data indicates sex differences in the mechanisms affected and in the responsiveness of male and female reproductive tracts by the alterations imposed by the hypothyroidism (**Table 4**), which opens an exciting field for future comparative investigations.

In terms of melatonin synthesis, the pineal gland plays a crucial role in synthesizing and releasing melatonin, a hormone closely associated with the light-dark cycle (63, 64). We observed different results in the gene expression of *Aanat* and *Asmt* in the pineal gland between males and females and at different time points. It is known that at ZT18, the animals are in the dark phase, and there is an endogenous increase in *Aanat* expression, which indicates higher pineal gland activity during this time (65). Therefore, the observed decrease in *Aanat* expression in males suggests reduction in the activity of the pineal gland in hypothyroid rats during these specific time points. Conversely, the reduction of *Asmt* expression at ZT18 was only noticed in pineal gland of hypothyroid female rats. The enzyme ASMT is considered a rate-limiting enzyme, as it is the final enzyme in the biosynthetic pathway responsible for converting N-acetylserotonin into melatonin in the pineal gland (66, 67). Therefore, a reduction in *Asmt* expression may indicate lower activity of this enzyme and, consequently, a decrease in melatonin synthesis. However, no significant reduction in plasma melatonin was observed in PTU-treated animals. This result contradicts other studies in Sprague-Dawley rats, where hypothyroidism was induced by intraperitoneal administration of PTU for 4 weeks, and a reduction in plasma melatonin levels was observed (37).

In males, this apparent disparity might be explicable by the steady expression of *Asmt* at ZT18, which offsets the reduction in N-acetylserotonin. However, a reduction in female plasma melatonin levels would be anticipated, considering ASMT’s role as the limiting rate-determining enzyme. In this context, an examination of other Zeitgeber Times (ZTs) would be worthwhile, as the reduced *Asmt* expression at ZT18 might exert a noteworthy impact on circulating melatonin levels later within the dark phase. In a previous study, ASMT in the pineal gland was detected throughout the 24-hour daily cycle, but a significant diurnal rhythm was not detected (66).

Regarding the gene expression of melatonin synthesizing enzymes in the gonads, our findings revealed an upregulation in the *Asmt* gene expression, particularly in animals euthanized at ZT11.5, in both ovaries and testicles. This result raises interesting questions about the role of melatonin in these structures, particularly in the context of hypothyroidism. The expression of *Asmt* in gonads is associated with melatonin synthesis, and the observed increase suggests higher activity of this enzyme in the synthesis of this hormone in the gonads at this specific time (ZT11.5) (11–13, 68, 69). The investigation of the melatonergic system in the reproductive tract of rats with hypothyroidism is underexplored. As of now, aside from our research group, no other studies have been identified to investigate *Aanat* and *Asmt* gene expression in the context of hypothyroidism in the reproductive organs.

Additionally, a negative correlation was observed between FT4 levels and *Asmt* gene expression in the ovaries, suggesting that hypothyroidism could increase the expression of the *Asmt* gene in the gonads. This relationship becomes interesting in the context of hypothyroidism, as levels of oxidative stress and apoptosis increase in the ovary (70), while local melatonin synthesis could be induced by oxidative stress and plays an antioxidant role, reducing these damages in ovarian tissue (21).

Regarding males, a negative correlation was observed between FT4 levels and *Asmt* gene expression in the gonads, indicating that in the context of hypothyroidism, the testicles tend to increase *Asmt* gene expression. Although this analysis is not causal, but it is an intriguing relationship. Especially considering that during hypothyroidism, there is an increase in oxidative stress in the testis, while melatonin has been associated with reducing such damage in this organ (31, 70).

It’s noteworthy that a negative interaction was also detected between male body weight and melatonin biosynthesis enzyme gene expression, both in the pineal gland and gonads, as well as the relationship between prostate weight and *Asmt* gene expression in the testes. Additionally, previous studies have reported a decreased prostate weight following treatment with a dose of melatonin (71, 72). On the other hand, the remaining correlations exhibited a positive interaction. It is important to emphasize that the mentioned correlations do not necessarily imply a direct causal relationship between the variables but rather indicate a statistical association among them (73).

The positive correlations between *Asmt* gene expression in the testes with plasma melatonin, and *Aanat* gene expression in pineal gland, as well as the negative correlation between *Asmt* gene expression in the testes, suggest that melatonin synthesis in testes could be modulated by hypothyroidism and pineal gland melatonin synthesis.

Our findings highlight the complexity of gene expression patterns of melatonin-synthesizing enzymes in the pineal gland and gonads, which are influenced by hormonal factors, environmental cues, sexual differences, and pathophysiological contexts. Additionally, it is important to note that melatonin synthesis could be modulated according to the physiological context (74–77).

It is important to highlight that the results of this study were obtained from animals subjected to a 15-day period of hypothyroidism, characterized as the acute phase of the disease. Despite the short duration, alterations in morphological parameters and components of the melatonergic system were observed. The variation in *Aanat* and *Asmt* gene expression between male and female rats reaffirms the impact of sex differences on melatonin circadian profiles (78). Remarkably, reduced expression was observed at ZT18, during the dark phase when gene expression levels are elevated. This raises speculation that a longer induction period could potentially affect even the plasma melatonin synthesis.

The expression of *Aanat* and *Asmt* genes in the gonads showed a similar pattern in both sexes, with an increase only at ZT11.5. We hypothesize that these gonads are influenced by local oxidative stress, which gives the tendency of hypothyroidism to enhance oxidative stress in ovarian and testicular tissues (70, 79–81), and this expression correlates with the transition from the light to the dark phase of the circadian cycle. This observation prompts the need for further in-depth investigations in this specific aspect of transition.

In conclusion, our findings indicate the modulation of the melatonergic system in the reproductive tracts of male and female rats during the early phase of hypothyroidism and provide more information to understand the relationship between thyroid function, melatonin synthesis, and reproductive organs in rats. These findings highlight the importance of the interaction between the thyroid and melatonin synthesis in the context of reproduction. However, it is essential to emphasize the need for further investigations to fully explore these associations. Future studies could focus on elucidating the underlying molecular mechanisms and deepening the analysis of these relationships in other animal models and humans. Expanding this research can lead to significant advancements in understanding reproductive disorders, enabling the development of more effective therapies, and promoting improvements in reproductive health for humans and other species.

## Data availability statement

The raw data supporting the conclusions of this article will be made available by the authors, without undue reservation.

## Ethics statement

The animal study was approved by Ethics Committee on the Use of Animals of Santa Cruz State University (UESC) (Protocol 26/2021). The study was conducted in accordance with the local legislation and institutional requirements.

## Author contributions

RP: Formal analysis, Investigation, Methodology, Writing – original draft, Writing – review & editing. PM: Investigation, Methodology, Writing – review & editing. BB: Investigation, Methodology, Writing – review & editing. JS: Investigation, Writing – review & editing. MF: Investigation, Writing – review & editing. YF: Investigation, Writing – review & editing. LS: Formal analysis, Writing – review & editing, Investigation, Methodology. RM: Conceptualization, Resources, Writing – review & editing, Formal analysis, Writing – original draft. PF: Conceptualization, Investigation, Methodology, Resources, Writing – review & editing, Funding acquisition. JS: Conceptualization, Formal analysis, Investigation, Methodology, Resources, Supervision, Writing – original draft, Writing – review & editing. ET: Conceptualization, Data curation, Formal analysis, Funding acquisition, Investigation, Methodology, Project administration, Resources, Supervision, Validation, Visualization, Writing – original draft, Writing – review & editing.
