# Supplementary material for: Effects of acute hypothyroidism on plasma melatonin and Aanat and Asmt expression in the pineal gland and gonads of rats
Source: Front Endocrinol (Lausanne). 2024 Aug 1;15:1322867. doi: 10.3389/fendo.2024.1322867 (PMC11324505; doi:10.3389/fendo.2024.1322867)
Supplement: Supplementary file 1 [file Table_1.docx]

**Supplementary Materials**

**Table 1S – Comparison of Thyroid Function, Anthropometric Measurements, and Morphological Parameters between Control and Hypothyroid Groups of Female Rats**

| **Parameter** |  |  | **Control** | **Hypothyroid** |
| --- | --- | --- | --- | --- |
| **Free thyroxine (ng/dL)** | | **Total** | 2.070 ± 0.087 | **0.218 ± 0.018*** |
|  |  | ZT 18 | 1.883 ± 0.090 | **0.176 ± 0.030*** |
|  |  | ZT 6 | 2.335 ± 0.186 | **0.258 ± 0.029*** |
|  |  | ZT 11.5 | 1.992 ± 0.110 | **0.215 ± 0.032*** |
| **Body weight (g)** | | **Total** | 216.5 ± 1.417 | 214.9 ± 1.581 |
|  |  | ZT 18 | 219.7 ± 2.459 | 214.3 ± 2.654 |
|  |  | ZT 6 | 215.8 ± 2.023 | 218.7 ± 2.929 |
|  |  | ZT 11.5 | 213.9 ± 2.619 | 211.8 ± 2.236 |
| **Weight of sexual organs (mg)** | **Ovary** | **Total** | 0.035 ± 0.001 | 0.032 ± 0.001 |
|  |  | ZT 18 | 0.036 ± 0.002 | 0.030 ± 0.002 |
|  |  | ZT 6 | 0.034 ± 0.003 | 0.034 ± 0.003 |
|  |  | ZT 11.5 | 0.034 ± 0.002 | 0.031 ± 0.002 |
|  | **Uterus** | **Total** | 0.280 ± 0.021 | 0.238 ± 0.014 |
|  |  | ZT 18 | 0.281 ± 0.037 | 0.244 ± 0.025 |
|  |  | ZT 6 | 0.272 ± 0.050 | 0.219 ± 0.024 |
|  |  | ZT 11.5 | 0.286 ± 0.029 | 0.250 ± 0.027 |
| **Histomorphometry** | **Primary follicle (n°)** | **Total** | 5.167 ± 0.185 | **4.278 ± 0.331*** |
|  |  | ZT 18 | 5.000 ± 0.365 | 4.167 ± 0.542 |
|  |  | ZT 6 | 4.833 ± 0.307 | 3.833 ± 0.749 |
|  |  | ZT 11.5 | 5.667 ± 0.210 | 4.833 ± 0.401 |
|  | **Secondary follicle (n°)** | **Total** | 2.611 ± 0.281 | 2.278 ± 0.225 |
|  |  | ZT 18 | 2.667 ± 0.333 | 2.333 ± 0.494 |
|  |  | ZT 6 | 2.333 ± 0.614 | 2.000 ± 0.365 |
|  |  | ZT 11.5 | 2.833 ± 0.542 | 2.500 ± 0.341 |
|  | **Antral follicle (n°)** | **Total** | 10.78 ± 0.985 | **7.389 ± 0.829*** |
|  |  | ZT 18 | 9.667 ± 0.714 | 8.667 ± 1.626 |
|  |  | ZT 6 | 11.50 ± 1.928 | 6.667 ± 1.406 |
|  |  | ZT 11.5 | 11.17 ± 2.301 | 6.833 ± 1.376 |
|  | **Corpus luteum (n°)** | **Total** | 1.056 ± 0.248 | 0.722 ± 0.135 |
|  |  | ZT 18 | 0.833 ± 0.307 | 0.500 ± 0.223 |
|  |  | ZT 6 | 1.000 ± 0.447 | 0.833 ± 0.166 |
|  |  | ZT 11.5 | 1.333 ± 0.557 | 0.833 ± 0.307 |
|  | **Myometrium (μm)** | **Total** | 341.2 ± 17.95 | 296.7 ± 21.22 |
|  |  | ZT 18 | 390.4 ± 23.56 | 338.5 ± 38.92 |
|  |  | ZT 6 | 303.2 ± 23.97 | 264.2 ± 43.10 |
|  |  | ZT 11.5 | 330.2 ± 36.87 | 287.3 ± 25.36 |
|  | **Endometrium (μm)** | **Total** | 542.6 ± 35.21 | 457.3 ± 24.18 |
|  |  | ZT 18 | 491.1 ± 55.43 | 436.6 ± 47.30 |
|  |  | ZT 6 | 613.5 ± 68.31 | 483.8 ± 51.47 |
|  |  | ZT 11.5 | 523.2 ± 57.43 | 451.5 ± 29.08 |

*****p<0.05 versus respective control group.

**Table 2S – Comparison of Thyroid Function, Anthropometric Measurements, and Morphological Parameters between Control and Hypothyroid Groups of Male Rats**

| **Parameter** |  |  | **Control** | **Hypothyroid** |
| --- | --- | --- | --- | --- |
| **Free thyroxine (ng/dL)** | | **Total** | 2.103 ± 0.084 | **0.255 ± 0.022*** |
|  |  | ZT 18 | 1.892 ± 0.129 | **0.304 ± 0.047*** |
|  |  | ZT 6 | 2.336 ± 0.187 | **0.278 ± 0.037*** |
|  |  | ZT 11.5 | 2.083 ± 0.079 | **0.193 ± 0.017*** |
| **Body weight (g)** | | **Total** | 304.9 ± 7.916 | **286.7 ± 6.113*** |
|  |  | ZT 18 | 285.2 ± 5.152 | **267.6 ± 2.405*** |
|  |  | ZT 6 | 334.2 ± 15.08 | 307.1 ± 13.86 |
|  |  | ZT 11.5 | 295.4 ± 10.88 | 285.4 ± 5.145 |
| **Weight of sexual organs (mg)** | **Testicle** | **Total** | 1.445 ± 0.024 | 1.384 ± 0.024 |
|  |  | ZT 18 | 1.502 ± 0.035 | **1.335 ± 0.030 *** |
|  |  | ZT 6 | 1.436 ± 0.017 | 1.451 ± 0.052 |
|  |  | ZT 11.5 | 1.397 ± 0.057 | 1.365 ± 0.032 |
|  | **Epididymis** | **Total** | 0.155 ± 0.006 | 0.136 ± 0.007 |
|  |  | ZT 18 | 0.159 ± 0.010 | **0.130 ± 0.006*** |
|  |  | ZT 6 | 0.173 ± 0.006 | 0.166 ± 0.010 |
|  |  | ZT 11.5 | 0.133 ± 0.013 | 0.113 ± 0.007 |
|  | **Seminal vesicle** | **Total** | 1.041 ± 0.073 | 0.995 ± 0.075 |
|  |  | ZT 18 | 1.203 ± 0.110 | 1.060 ± 0.101 |
|  |  | ZT 6 | 1.206 ± 0.072 | 1.114 ± 0.186 |
|  |  | ZT 11.5 | 0.715 ± 0.079 | 0.813 ± 0.052 |
|  | **Prostate** | **Total** | 0.365 ± 0.022 | **0.268 ± 0.021*** |
|  |  | ZT 18 | 0.408 ± 0.030 | **0.259 ± 0.023*** |
|  |  | ZT 6 | 0.420 ± 0.023 | 0.337 ± 0.042 |
|  |  | ZT 11.5 | 0.268 ± 0.030 | 0.210 ± 0.029 |
| **Histomorphometry** | **Seminiferous tubule - height (μm)** | **Total** | 33.92 ± 1.088 | 32.30 ± 0.941 |
|  |  | ZT 18 | 31.42 ± 1.515 | 30.20 ± 1.334 |
|  |  | ZT 6 | 37.38 ± 1.666 | 34.14 ± 1.692 |
|  |  | ZT 11.5 | 32.96 ± 1.781 | 32.58 ± 1.669 |
|  | **Seminiferous tubule - diameter (μm)** | **Total** | 244.4 ± 13.01 | 253.5 ± 3.702 |
|  |  | ZT 18 | 215.5 ± 36.97 | 265.0 ± 4.713 |
|  |  | ZT 6 | 255.5 ± 4.720 | 247.4 ± 5.532 |
|  |  | ZT 11.5 | 262.1 ± 9.184 | 248.3 ± 6.909 |

*p<0.05 versus respective control group.
